# Efficient Prediction and Analysis of Optical Trapping at Nanoscale via Finite Element Tearing and Interconnecting Method

**DOI:** 10.1186/s11671-019-3131-7

**Published:** 2019-08-27

**Authors:** Ting Wan, Benliu Tang

**Affiliations:** 1 College of Telecommunications and Information Engineering, Nanjing University of Posts and Telecommunications, Nanjing, 210003 China; 2 State Key Laboratory of Millimeter Waves, Nanjing, 210096 China

**Keywords:** Optical trapping, Optical force, Nanoparticle, Surface plasmon, Numerical simulation

## Abstract

Numerical simulation plays an important role for the prediction of optical trapping based on plasmonic nano-optical tweezers. However, complicated structures and drastic local field enhancement of plasmonic effects bring great challenges to traditional numerical methods. In this article, an accurate and efficient numerical simulation method based on a dual-primal finite element tearing and interconnecting (FETI-DP) and Maxwell stress tensor is proposed, to calculate the optical force and potential for trapping nanoparticles. A low-rank sparsification approach is introduced to further improve the FETI-DP simulation performance. The proposed method can decompose a large-scale and complex problem into small-scale and simple problems by using non-overlapping domain division and flexible mesh discretization, which exhibits high efficiency and parallelizability. Numerical results show the effectiveness of the proposed method for the prediction and analysis of optical trapping at nanoscale.

## Introduction

Plasmonic optical tweezers based on surface plasmons (SPs) draw much attention and have been widely applied to capture nanoparticles [1–6]. SP is a resonance phenomenon caused by the coupling of incident light with a specific wavelength and free electrons at the interface of the metals and dielectrics [7]. SPs enable the optical tweezers to break through the diffraction limit. Moreover, the drastic local field enhancement of the SPs can reduce the demand of intensity of incident light [7, 8]. However, SPs are closely related to the material and dimensions of objects as well as the wavelength of incident light, which requires a large number of experiments to determine the optimal parameters of SP optical tweezers in practice. Based on this, the simulation method plays an increasingly important role as an auxiliary mean for the design and optimization of SP optical tweezers [9]. In these simulations, the calculation of optical force is required to predict a stable trapping. For regular objects such as spheres, the optical force can be analytically derived from generalized Lorenz-Mie theory [10, 11]. However, for objects with complicated configurations, numerical methods that solve the governing Maxwell’s equations rigorously are necessary for modeling the electromagnetic fields and the subsequent optical force and potential.

These numerical methods can be mainly categorized into differential equation methods (DEMs) and integral equation methods (IEMs) [12–15]. Compared with the IEMs, differential equation methods (DEMs) show superior abilities in dealing with complicated geometries and components. DEMs also have the advantage of a straightforward calculation of near-field distribution, which plays an important role in the analysis of SPs. As a representative DEM, finite-difference time-domain (FDTD) method is implemented in the time domain, which can easily get broadband information and transient responses [16, 17]. However, the FDTD demands an accurate dispersive model to describe the frequency-dependent material properties in SPs, while the FDTD solution accuracy highly depends on the approximation accuracy of this dispersive model [18]. Besides, the FDTD relies on structured meshes, which often lead to staircase error for curved surfaces. As another representative DEM, finite element method (FEM) has been widely adopted since it can easily handle dispersive material in the frequency domain and eliminate the staircase error by unstructured mesh [19–22]. Compared with the FDTD, the FEM can directly adopt measured material parameters without any approximate dispersive model. However, drastic local field enhancements in the SPs require fine meshes in the FEM discretization. Besides, objects with large dimensions and multiple objects will dramatically increase the number of unknowns. These factors will cause ill-conditioned matrix systems and huge computational consumptions, which bring great challenges to traditional FEM for the analysis of SP-enhanced optical trapping.

In this article, an efficient dual-primal finite element tearing and interconnecting (FETI-DP) method is introduced to simulate the optical trapping at nanoscale. The FETI-DP adopts a non-overlapping domain decomposition scheme, which divides an original large-scale complex problem into a series of small-scale simple problems to conquer them. It enforces a transmission condition at the subdomain interfaces to ensure the filed continuity, and introduces a dual variable to reduce original three-dimensional (3D) problem to be a two-dimensional (2D) problem by Lagrange multiplier. Primal variables at the subdomain corners are extracted to accelerate the convergence rate of iterative solution of the dual problem [23–26]. A low-rank sparsification approach is developed to improve the performance of the FETI-DP. It uses data-sparse algorithms to improve the efficiency for solving the subdomain problems and the dual problem [27, 28]. The proposed method provides fully decoupled subdomains, which enable the parallel simulation of optical force for trapping nanoparticles. The Maxwell stress tensor (MST) that reveals the relationship between the electromagnetic field and mechanical momentum is adopted to evaluate the optical force [29]. Based on the obtained optical force, the optical potential can be further calculated for the analysis of a stable trapping. Compared with the IEMs, the proposed method is more powerful in dealing with compound materials and solving the near-field for the SP-based optical trapping. Compared with the FDTD, the proposed method can accurately handle dispersive metal material in the SP-based optical trapping systems and eliminate the staircase error for the objects with curve boundary. Compared with the FEM, the proposed method is suitable for large-scale computation of optical trapping. Several examples are analyzed and numerical results demonstrate the accuracy and efficiency of the proposed method for the prediction and analysis of optical trapping at nanoscale.

## Methods

### FETI-DP Formulations

**Fig. 1:**
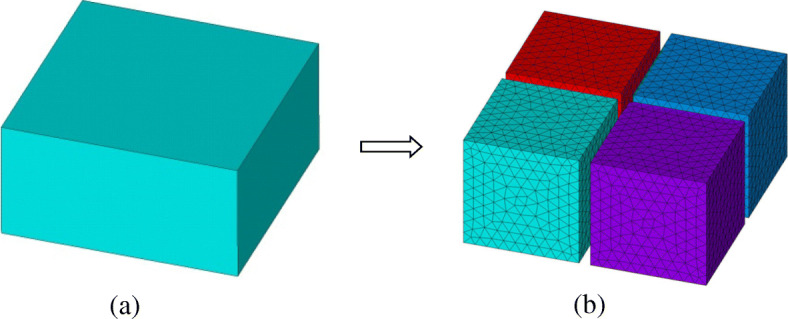
A domain division scheme with non-overlapping subdomains in the FETI-DP method. **a** Original domain. **b** Divided subdomains and discretized meshes

For the FETI-DP implementation, the original computational domain Ω is first torn into a series of non-overlapping subdomains Ω^*i*^ (*i* = 1, 2, 3⋯, *N*_*s*_), as shown in Fig. 1. In each subdomain Ω^*i*^, a subdomain finite element system can be derived from the vector wave equation as

1 $$ \nabla \times {\mu}_r^{-1}\nabla \times {\mathbf{E}}^i-{k}_0^2{\varepsilon}_r{\mathbf{E}}^i={jk}_0{\eta}_0{\mathbf{J}}_{\mathrm{imp}}^i\kern1.08em \mathrm{in}\kern0.24em {\Omega}^i $$

2 $$ \hat{n}\times \nabla \times {\mathbf{E}}^i+{jk}_0\hat{n}\times \left(\hat{n}\times {\mathbf{E}}^i\right)=0\kern0.96em \mathrm{on}\kern0.24em {\Gamma}_{\mathrm{ABC}}^i $$

where **E**^*i*^ denotes the unknown electric field to be solved in ${\Omega}^i$, *k*_0_ and *η*_0_ are the free-space wavenumber and intrinsic impedance, respectively, and ${\mathbf{J}}_i^{\mathrm{imp}}$ is the impressed current. ${\Gamma}_{\mathrm{ABC}}^i$ means the absorbing boundary condition (ABC) to truncate the infinite open region. It should be noted that *k*_0_ should be replaced by the wave impedance in medium if the surrounding medium is not free-space, which is common for the optical trapping. At the subdomain interface Γ^*i*^, an assumed boundary condition is required to generate a complete boundary value problem in Ω^*i*^. Here, a Robin-type transmission condition with an unknown auxiliary variable **Λ**_*i*_ is imposed as

3 $$ {\hat{n}}^i\times \left({\mu}_r^{-1}\nabla \times {\mathbf{E}}^i\right)+{\alpha}^i{\hat{n}}^i\times \left({\hat{n}}^i\times {\mathbf{E}}^i\right)={\boldsymbol{\Lambda}}^i\kern1.2em \mathrm{on}\kern0.36em {\Gamma}^i $$

where ${\hat{n}}^i$ denotes unit normal outward vector at the subdomain interface Γ^*i*^, and *α*^*i*^ is a complex parameter which can often be chosen as *jk*_0_. All subdomains are then discretized by tetrahedral elements. In each element, we expand **E** with vector basis functions **N** and unknown electric field coefficient *E* as

4 $$ \mathbf{E}=\sum \limits_{p=1}^s{E}_p{\mathbf{N}}_p $$

where *s* denotes the number of vector basis functions in each tetrahedral element. *s* is chosen to be 6 for traditional low-order basis function based on the edge, while it is larger for high-order vector basis function, since additional basis functions based on face or volume are introduced.

Applying Galerkin’s method, the FEM matrix equation in Ω^*i*^ about the unknown electric field coefficient *E*^*i*^ can be obtained as

5 $$ \left(\begin{array}{cc}{\mathbf{K}}_{rr}^i& {\mathbf{K}}_{rc}^i\\ {}{\mathbf{K}}_{cr}^i& {\mathbf{K}}_{cc}^i\end{array}\right)\left(\begin{array}{l}{E}_r^i\\ {}{E}_c^i\end{array}\right)=\left(\begin{array}{l}{f}_r^i-{\mathbf{B}}_r^{i^T}{\lambda}^i\\ {}{f}_c^i\end{array}\right) $$

where the subscript notations *c* and *r* distinguish the corner degrees of freedom (DOFs) and the remaining DOFs, which extracts the corner DOFs as a primal variable to construct the dual-primal (DP) scheme. Here, **K** is the FEM system matrix and *f* is the excitation vector. **B** is a Boolean matrix that extracts the interface DOFs of a subdomain. *λ* is a dual variable generated from expanding **Λ**_*i*_, which is also called the Lagrange multiplier.

Then, the adjacent subdomains can be interconnected by enforcing tangential electric field and magnetic field continuity at the interfaces. We assemble all subdomain interfaces and eliminate all the subdomain internal unknowns *E*^*i*^. A reduced global interface equation about the dual variable *λ* can be obtained as

6 $$ \left[{\tilde{\mathbf{K}}}_{rr}+{\tilde{\mathbf{K}}}_{rc}{\tilde{\mathbf{K}}}_{cc}^{-1}{\tilde{\mathbf{K}}}_{cr}\right]\lambda ={\tilde{f}}_r-{\tilde{\mathbf{K}}}_{rc}{\tilde{\mathbf{K}}}_{cc}^{-1}{\tilde{f}}_c $$

Equation (6) can be solved by iterative methods, such as the generalized minimal residual (GMRES) method. ${\tilde{\mathbf{K}}}_{cc}$ is the global corner system, which can accelerate the iterative convergence in primal space. Suitable preconditioner can be employed to improve iterative convergence rate, such as approximate inverse and incomplete LU decomposition. Once the dual variable *λ* is solved, the electric field inside each subdomain can be independently evaluated by (5). For the construction of the global matrix ${\tilde{\mathbf{K}}}_{rr}$, one needs to construct the subdomain numerical Green’s function ${\mathbf{Z}}_{rr}^i$ with a form of

7 $$ {\mathbf{Z}}_{rr}^i={\mathbf{B}}_r^i{\left({\mathbf{K}}_{rr}^i\right)}^{-1}{{\mathbf{B}}_r^i}^T $$

where the inverse of the subdomain FEM matrix ${\left({\mathbf{K}}_{rr}^i\right)}^{-1}$ is included. Besides, for matrices ${\tilde{\mathbf{K}}}_{rc}$, ${\tilde{\mathbf{K}}}_{cr}$, and ${\tilde{\mathbf{K}}}_{cc}$ and vectors ${\tilde{f}}_r$ and ${\tilde{f}}_c$, the ${\left({\mathbf{K}}_{rr}^i\right)}^{-1}$ is required to be computed. The constructions of ${\left({\mathbf{K}}_{rr}^i\right)}^{-1}$ at pre-processing stage as well as their matrix-vector products (MVPs) at iterative solution stage are computationally expensive. Although ${\mathbf{K}}_{rr}^i$ is sparse, ${\left({\mathbf{K}}_{rr}^i\right)}^{-1}$ are dense, which means high computational costs. Next, a low-rank sparsification method is introduced to accelerate the computation of ${\left({\mathbf{K}}_{rr}^i\right)}^{-1}$. Since some sub-matrices in the global interface system can be represented in low-rank matrix form, their computation can be performed with low-rank algorithm, which improves the performance of the FETI-DP. It can be seen that the FETI-DP provides independent subdomain operations, so that it can exploit parallel computation to improve efficiency. For an efficient parallel scheme, a principle of domain division is to make the number of DOFs in each subdomain as balanced as possible. Hence, the size of subdomains should relate to the mesh discretization density. Usually, small subdomains are adopted in finely meshed areas, while large subdomains are adopted in coarsely meshed areas.

### Low-Rank Sparsification

A low-rank sparsification approach is proposed to provide a data-sparse way to improve the FETI-DP efficiency. Here, *data-sparse* means these matrices are actually not sparse but they are sparse in the sense that certain sub-blocks of them can be represented by low-rank decomposition matrix forms as

8 $$ \mathbf{M}={\mathbf{XY}}^{\mathrm{T}}\kern0.72em \left(\mathbf{M}\in {\mathrm{\mathbb{C}}}^{m\times n},\mathbf{X}\in {\mathrm{\mathbb{C}}}^{m\times k},\mathbf{Y}\in {\mathrm{\mathbb{C}}}^{n\times k}\right) $$

where **X** and **Y** are in full matrix forms, and the rank *k* is much smaller than *m* and *n*. The matrix ${\left({\mathbf{K}}_{rr}^i\right)}^{-1}$ can be represented by data-sparse matrix forms since it possesses the matrix property of an integral operator. Hence, provided ${\left({\mathbf{K}}_{rr}^i\right)}^{-1}$ possess low-rank property in given subdomain, it can be efficiently computed and stored in data-sparse forms with the low-rank sparsification approach, which accelerates the MVPs in the iterative solution.

The processes of the low-rank sparsification approach can be divided into the following steps: (1) construct a cluster tree by subdividing the basis function set in each subdomain, (2) construct a block cluster tree by interaction of two cluster trees, (3) generate a data-sparse form of ${\mathbf{K}}_{rr}^i$ by an admissibility condition, (4) perform low-rank formatted algorithms to get the data-sparse representation of ${\left({\mathbf{K}}_{rr}^i\right)}_{\mathrm{DS}}^{-1}$, and (5) enter the solution of FETI-DP systems by data-sparse algorithm. Suitable preconditioner can be employed to speed up the solution. It should be noted that the data-sparse LU factorization ${\left({\mathbf{K}}_{rr}^i\right)}_{\mathrm{DS}}={\left({\mathbf{L}}_{rr}^i\right)}_{\mathrm{DS}}{\left({\mathbf{U}}_{rr}^i\right)}_{\mathrm{DS}}$ is adopted to replace the matrix inversion ${\left({\mathbf{K}}_{rr}^i\right)}_{\mathrm{DS}}^{-1}$. A nested dissection technique is employed to further improve the efficiency of the low-rank sparsification. The nested dissection uses separators to yield large off-diagonal zero sub-blocks, which will keep zero during the LU factorization so that it can significantly reduce the fill-ins.

**Fig. 2:**
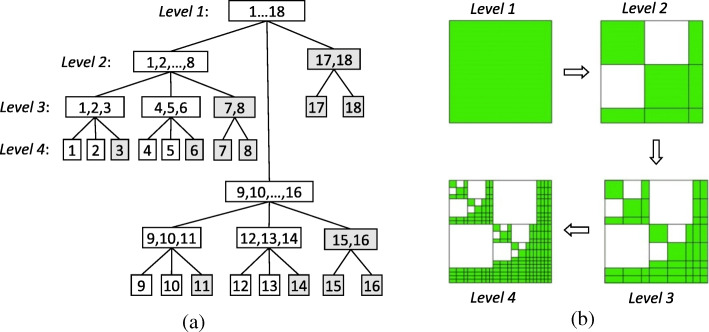
Constructions of a cluster tree and a block cluster tree of 4 levels based on nested dissection. **a** Construction of a cluster tree by recursive subdivision of edge-based basis function set *I* = {1,2,…18}. **b** Construction of a block cluster tree where *white* blocks are zero matrices and *green* blocks can be full matrices or low-rank decomposition matrices

To generate ${\left({\mathbf{K}}_{rr}^i\right)}_{\mathrm{DS}}$, we first construct a cluster tree *T*_*I*_ by recursive subdivision of the subdomain edge-based basis function set *I* = {1,2,……*N*} using bounding box. With the nested dissection, a cluster *t* within the corresponding bounding box is divided into three successors {*s*_1_, *s*_*sep*_, *s*_2_}, where *s*_1_ and *s*_2_ are the index sets of the two disconnected bounding boxes and *s*_*sep*_ is the index set of the separator. Figure 2 a shows a simple example of this process. Then, a block cluster tree *T*_*I* × *I*_ can be constructed by interacting two cluster tree *T*_*I*_, as shown in Fig. 2 b, which can be chosen as the cluster tree of the original edge-based basis function set and that of the testing basis function set in Galerkin’s method. Next, we need to introduce an admissibility condition based on the nested dissection to distinguish full blocks, low-rank decomposition blocks and off-diagonal zero blocks in *T*_*I* × *I*_ [23]. Thus, ${\left({\mathbf{K}}_{rr}^i\right)}_{\mathrm{DS}}$ can be produced by filling the corresponding blocks with the non-zero entries of ${\mathbf{K}}_{rr}^i$. Finally, the data-sparse LU factorization of ${\left({\mathbf{K}}_{rr}^i\right)}_{\mathrm{DS}}={\left({\mathbf{L}}_{rr}^i\right)}_{\mathrm{DS}}{\left({\mathbf{U}}_{rr}^i\right)}_{\mathrm{DS}}$ can be calculated recursively from

9 $$ {\mathbf{K}}_{rr}^i=\left[\begin{array}{ccc}{\mathbf{K}}_{11}& & {\mathbf{K}}_{13}\\ {}& {\mathbf{K}}_{22}& {\mathbf{K}}_{23}\\ {}{\mathbf{K}}_{31}& {\mathbf{K}}_{32}& {\mathbf{K}}_{33}\end{array}\right]=\left[\begin{array}{ccc}{\mathbf{L}}_{11}& & \\ {}& {\mathbf{L}}_{22}& \\ {}{\mathbf{L}}_{31}& {\mathbf{L}}_{32}& {\mathbf{L}}_{33}\end{array}\right]\left[\begin{array}{ccc}{\mathbf{U}}_{11}& & {\mathbf{U}}_{13}\\ {}& {\mathbf{U}}_{22}& {\mathbf{U}}_{23}\\ {}& & {\mathbf{U}}_{33}\end{array}\right] $$

where conventional full matrix arithmetics are replaced by their data-sparse counterparts [28]. An adaptive truncation error *ε*_*t*_ is employed to control the accuracy of low-rank approximations. The obtained LU factors ${\left({\mathbf{L}}_{rr}^i\right)}_{\mathrm{DS}}$ and ${\left({\mathbf{U}}_{rr}^i\right)}_{\mathrm{DS}}$ are stored and used to construct ${\mathbf{Z}}_{rr}^i$ by

10 $$ {\mathbf{Z}}_{rr}^i={\mathbf{B}}_r^i{\left({\mathbf{U}}_{rr}^i\right)}_{\mathrm{DS}}^{-1}{\left({\mathbf{L}}_{rr}^i\right)}_{\mathrm{DS}}^{-1}{\mathbf{B}}_r^i $$

where ${\mathbf{B}}_r^i{\left({\mathbf{U}}_{rr}^i\right)}_{\mathrm{DS}}^{-1}$ and ${\left({\mathbf{L}}_{rr}^i\right)}_{\mathrm{DS}}^{-1}{\mathbf{B}}_r^i$ can be computed by data-sparse upper and lower triangular solver. The ${\left({\mathbf{L}}_{rr}^i\right)}_{\mathrm{DS}}$, ${\left({\mathbf{U}}_{rr}^i\right)}_{\mathrm{DS}}$, and ${\mathbf{Z}}_{rr}^i$ enter the FETI-DP calculation with data-sparse forward and backward substitutions (FBSs) and data-sparse MVPs.

### Optical Force and Potential

According to electrodynamics theory, the optical force can be evaluated by the Maxwell stress tensor (MST) that reveals the relationship between electromagnetic field and mechanical momentum [29]. Once the electromagnetic field distribution around the object is obtained, the optical force can be calculated by integrating MST over a closed surface surrounding the object. Based on the obtained electric field distribution, the MST at any coordinates can be constructed by

11 $$ \overleftrightarrow{\mathbf{T}}=\frac{1}{2}\operatorname{Re}\left[\varepsilon {\mathbf{EE}}^{\ast }+\mu {\mathbf{HH}}^{\ast }-\frac{1}{2}\left(\varepsilon {\left|\mathbf{E}\right|}^2+\mu {\left|\mathbf{H}\right|}^2\right)\overleftrightarrow{\mathbf{I}}\right] $$

where the superscript asterisk denotes the conjugate of electric field or magnetic field, *ε* are *μ* are the permittivity and permeability, and $\overleftrightarrow{\mathbf{I}}$ is a 3 × 3 identity matrix. By the outer product of vectors, the tensor form of $\overleftrightarrow{\mathbf{T}}$ can be written as

12 $$ \overleftrightarrow{\mathrm{T}}=\left[\begin{array}{lll}{T}_{xx}& {T}_{xy}& {T}_{xz}\\ {}{T}_{yx}& {T}_{yy}& {T}_{yz}\\ {}{T}_{zx}& {T}_{zy}& {T}_{zz}\end{array}\right]=\left[\begin{array}{ccc}\varepsilon {E}_x{E}_x^{\ast }+\mu {H}_x{H}_x^{\ast }-\frac{\varepsilon {\left|\mathbf{E}\right|}^2+\mu {\left|\mathbf{H}\right|}^2}{2}& \varepsilon {E}_x{E}_y^{\ast }+\mu {H}_x{H}_y^{\ast }& \varepsilon {E}_x{E}_z^{\ast }+\mu {H}_x{H}_z^{\ast}\\ {}\varepsilon {E}_y{E}_x^{\ast }+\mu {H}_y{H}_x^{\ast }& \varepsilon {E}_y{E}_y^{\ast }+\mu {H}_y{H}_y^{\ast }-\frac{\varepsilon {\left|\mathbf{E}\right|}^2+\mu {\left|\mathbf{H}\right|}^2}{2}& \varepsilon {E}_y{E}_z^{\ast }+\mu {H}_y{H}_z^{\ast}\\ {}\varepsilon {E}_z{E}_x^{\ast }+\mu {H}_z{H}_x^{\ast }& \varepsilon {E}_z{E}_y^{\ast }+\mu {H}_z{H}_y^{\ast }& \varepsilon {E}_z{E}_z^{\ast }+\mu {H}_z{H}_z^{\ast }-\frac{\varepsilon {\left|\mathbf{E}\right|}^2+\mu {\left|\mathbf{H}\right|}^2}{2}\end{array}\right] $$

where the subscript *x*, *y*, *z* denotes the components in three directions. According to the expanding of **E** described in (4), the entries of MST *T*_*mn*_ (*m*, *n* = *x*, *y*, *z*) can be converted into expanded forms in the FETI-DP calculation as

13 $$ {\displaystyle \begin{array}{l}{T}_{mn}=\sum \limits_{p,q=1}^s{E}_p{E}_q\left\{\varepsilon {\left({\mathbf{N}}_p\right)}_m{\left({\mathbf{N}}_q^{\ast}\right)}_n-\frac{1}{\omega^2\mu }{\left(\nabla \times {\mathbf{N}}_p\right)}_m{\left(\nabla \times {\mathbf{N}}_q^{\ast}\right)}_n\right.\\ {}\kern1.75em \left.-\frac{1}{2}\left[\varepsilon \left({\mathbf{N}}_p\right)\left({\mathbf{N}}_q^{\ast}\right)-\frac{1}{\omega^2\mu}\left(\nabla \times {\mathbf{N}}_p\right)\left(\nabla \times {\mathbf{N}}_q^{\ast}\right)\right]\right\}\kern1.75em \mathrm{if}\ m=n.\end{array}} $$

14 $$ {T}_{mn}=\sum \limits_{p,q=1}^s{E}_p{E}_q\left\{\varepsilon {\left({\mathbf{N}}_p\right)}_m{\left({\mathbf{N}}_q^{\ast}\right)}_n-\frac{1}{\omega^2\mu }{\left(\nabla \times {\mathbf{N}}_p\right)}_m{\left(\nabla \times {\mathbf{N}}_q^{\ast}\right)}_n\right\}\kern1.25em \mathrm{if}\ m\ne n. $$

where *ω* is the angular frequency; **N** and *s* have been described in Eq. (4).

Finally, the optical force exerted on the object can be calculated by integrating the MST over any closed surface surrounding it by

15 $$ \mathbf{F}={\oint}_S\left(\overleftrightarrow{\mathbf{T}}\cdot \hat{n}\right)\  dS. $$

Note that the calculation of optical force can also be implemented in parallel, since the integral of the MST is assigned to corresponding subdomains. For a stable optical trapping, one of the main conditions is that the gradient force should be greater than the scattering force. In other words, the direction of the total force should be identical with that of the gradient force, which always points to the position where the electric field intensity is strongest.

The optical potential is another attractive parameter revealing the stability of the optical trapping. Based on the obtained optical force, the optical potential depth **U** at position *r*_0_ can be calculated by

16 $$ \mathbf{U}\left({r}_0\right)=-{\int}_{\infty}^{r_0}\mathbf{F}\left(\mathbf{r}\right)\cdot \mathbf{r}, $$

where the subscript ∞ denotes infinity defined as the reference point with zero potential. The value of **U** can be represented by *k*_*B*_T, where *k*_*B*_ denotes the Boltzmann constant of 1.3806488 × 10^−23^J/K and T is the ambient temperature. Generally, the particle can overcome the Brownian motion in solution and be stably trapped when **U** > 1 *k*_*B*_T is satisfied. Otherwise, the particle cannot be stably trapped. Since the total optical force includes the conservative gradient force component and the non-conservative scattering force component, the total optical force **F** from (15) is non-conservative [30, 31]. However, provided the motion of the nanoparticle is restricted to one dimension, this yields an unambiguous definition of an optical potential from (16), even though the total optical force is non-conservative.

## Results and Discussion

Three examples are presented to demonstrate the effectiveness of the proposed method. Since noble metals are commonly used to excite the surface plasmon, we select representative gold and silver materials for the analyses. The first example calculates the optical force of silver nanoparticle to verify the accuracy of the proposed method. The second and third examples simulate and discuss the optical trapping of gold nanoparticles. For all the examples, the infinite domain is truncated with ABC, and the distances between the ABC and the objects are set to be one wavelength, which is sufficient to achieve an acceptable accuracy. All calculations are performed on a Dell workstation equipped with 3.6 GHz Intel Xeon processors.

### Silver Nanocapsule

**Fig. 3:**
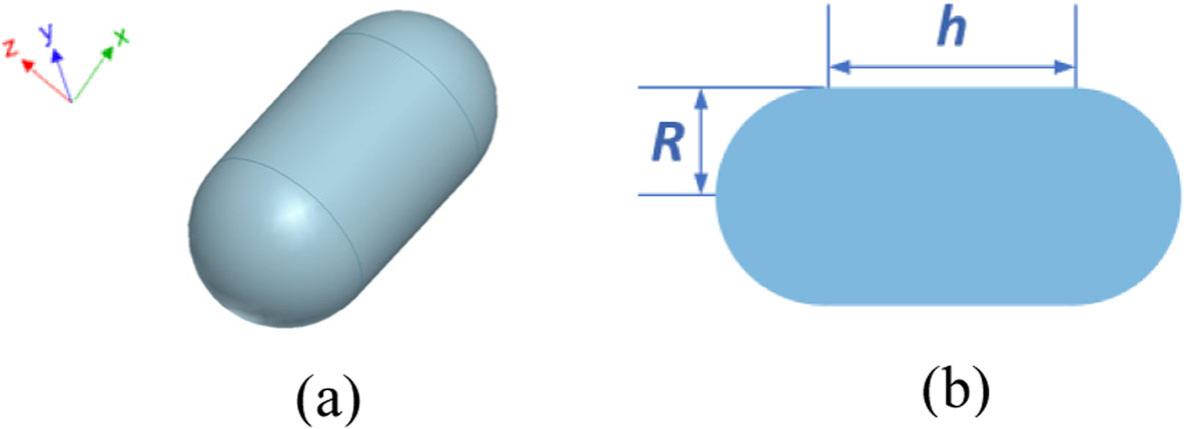
Configuration of a silver nanocapsule structure. **a** 3D view. **b** Front view and dimensions, where *R* = 30 nm and *h* = 60 nm

A silver nanocapsule object is first considered to test the accuracy and efficiency of the proposed FETI-DP method in predicting optical force. Figure 3 a and b presents its configuration and dimensions. The constitutive parameters of silver are all measured values taken from [32]. To implement the FETI-DP scheme, the whole analysis domain is first divided into 24 subdomains. Denser meshes are required near the metal surface in order to model the plasmonic local field enhancement effect. Tetrahedral elements are adopted for the discretization, which leads to totally 6.9 × 10^5^ unknowns, including 4.1 × 10^4^ dual unknowns and 313 corner unknowns. The incident light illuminates along the direction of +*z*, while the direction of polarization of electric field is −*x*.

**Fig. 4:**
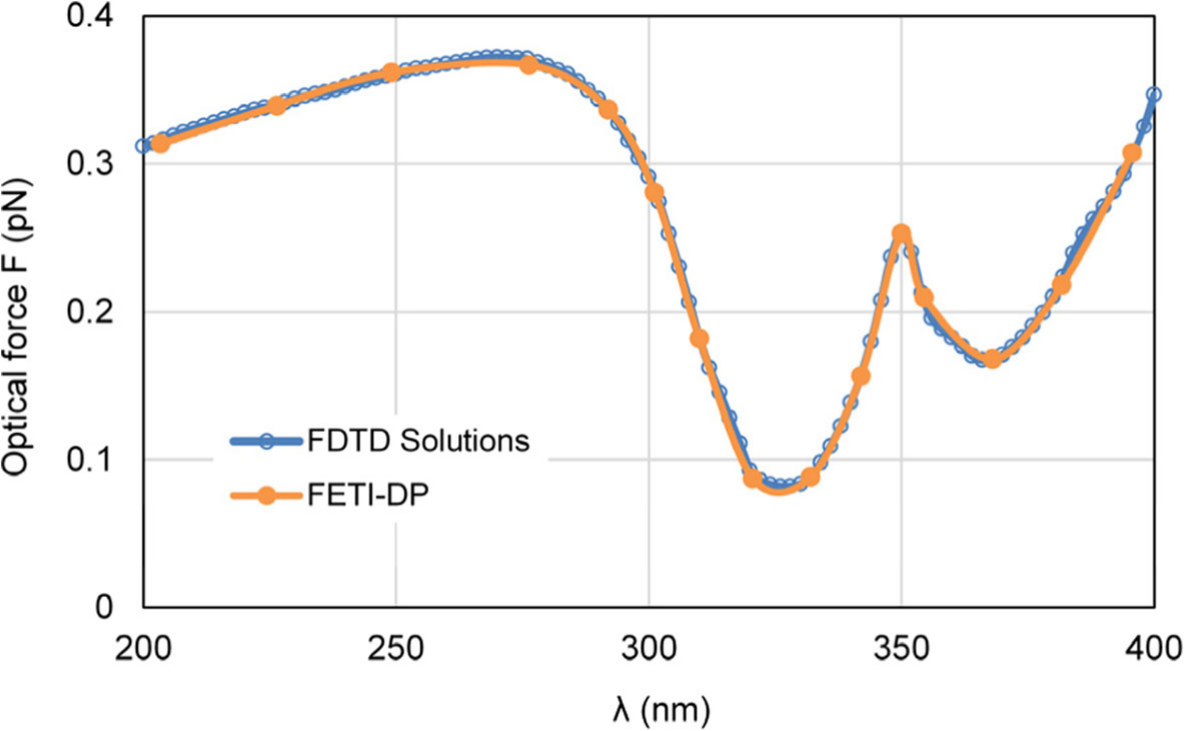
Results of the optical forces exerted on the silver nanocapsule, varying with the wavelength *λ* of incident light, including the results of the FETI-DP and the commercial software FDTD solutions

First, we change the wavelength of incident light *λ* from 200 nm to 400 nm to simulate the optical forces exerted on the nanocapsule. Since the FETI-DP works in frequency domain, the optical forces are calculated at 15 sampling frequency points. Figure 4 shows the calculated curve of optical forces exerted on the silver nanocapsule. To indicate the accuracy of the FETI-DP, the optical force results of the FETI-DP are compared with those of the commercial software Lumerical FDTD Solutions [33], and good agreement can be observed.

Then, the performance of FETI-DP is tested for different numbers of subdomains. We increase the number of subdomains from 4 to 24 by keeping the discretization density. We assign each processor to deal with one subdomain. Table 1 reports the time used for the construction of global interface Eq. (6) and the total solution time. It can be seen that the FETI-DP can fully exploit parallel computing resources and significantly improve the solution efficiency. Besides, the accuracy of the FETI-DP with the number of subdomains increasing is also examined and reported in Table 1. Here, the accuracy is defined by the 2-norm relative error of the optical force as *δ*_*OF*_ = ‖*OF*_*i*_ − *OF*_ref_‖/‖*OF*_ref_‖, where *OF*_*i*_ is the optical force using *i* subdomains and *OF*_ref_ denotes the reference optical force using two subdomains. It can be seen that the accuracy keeps almost constant with the number of subdomains increasing.

**Table 1:** Performance of the FETI-DP for calculating the optical force exerted on the silver nanocapsule with different number of subdomains

Number of subdomains	Construction time (s)	Total time (s)	Relative error *δ*_*OF*_
4	265.6	436.6	5.2 × 10^−4^
8	130.8	215.0	7.0 × 10^−4^
16	57.4	101.4	3.3 × 10^−4^
24	27.9	57.8	7.8 × 10^−4^

### Gold Nanosphere Dimer

**Fig. 5:**
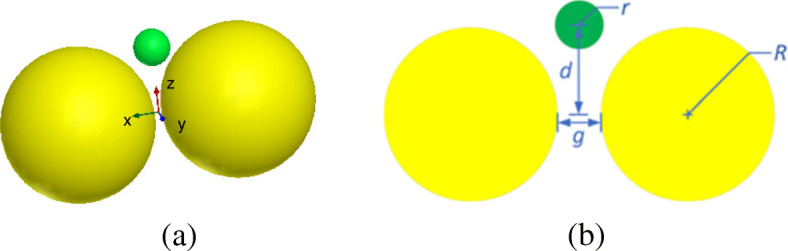
Configuration of an optical trapping system of a gold nansphere dimer in water. **a** 3D view. **b** Front view and dimensions, where *R* = 25 nm, *r* = 5 nm, and *g* = 2 nm

The second example analyzes the optical trapping of a gold nanosphere by using a gold nanosphere dimer. The plasmonic effects at the dimer gap can effectively enhance the optical force for trapping nanoparticle. Figure 5 a and b gives the configuration and dimensions of this system. The constitutive parameters of gold are all measured values taken from [32]. The surrounding medium is water with a relative refractive index of *n* = 1.33. The incident light is a plane wave with the power of 10 mW/μm^2^, the electric field polarization direction is +*x*, and the incident direction is −*z*. The optical force exerted on the object nanosphere is calculated by the FETI-DP method. For the FETI-DP implementation, the whole computational domain is divided into 32 subdomains and discretized by tetrahedral meshes, which results in 3.5 × 10^6^ unknowns, including 1.6 × 10^5^ dual unknowns and 1738 corner unknowns.

First, we test the parallel performance of the proposed FETI-DP by using various numbers of processors. Table 2 reports the solution time for Eq. (6) as well as the total solution time. Besides, the speedups for the parallel computation are also provided in Table 2. Here, the speedup is defined by

17 $$ \mathrm{Speed}\ \mathrm{up}=\raisebox{1ex}{${T}_1$}\!\left/ \!\raisebox{-1ex}{${T}_{N_p}$}\right. $$

**Table 2:** Time used for the FETI-DP and parallel speedup for calculating the optical force of the nanosphere dimer system with 32 subdomains and 3.5 million unknowns

Number of processors	Interface time (s)	Total time (s)	Speed up
1	1869	6672	1.0
4	595	1853	3.6
8	320	967	6.9
16	169	520	12.8
32	88	272	24.5

where ${T}_{N_p}$ denotes the total wall-clock time using *N*_*p*_ processors. It can be seen that the FETI-DP significantly improves the solution efficiency and exhibits good parallel speedup. For this large number of unknowns, the total memory usage of all the processors is only 57.2 GB.

Then, the effectiveness of the low-rank sparsification approach is examined. With the low-rank sparsification, the subdomain matrix can be factorized by data-sparse algorithm and stored as data-sparse matrices. The construction time and memory usage are only 18 s and 0.5 GB, while they are 67 s and 1.7 GB by conventional matrix algorithm. It can be seen that we get 72% time saving and 70% memory compression. Related to the memory usage, the subsequent MVPs can also get 70% time-saving.

**Fig. 6:**
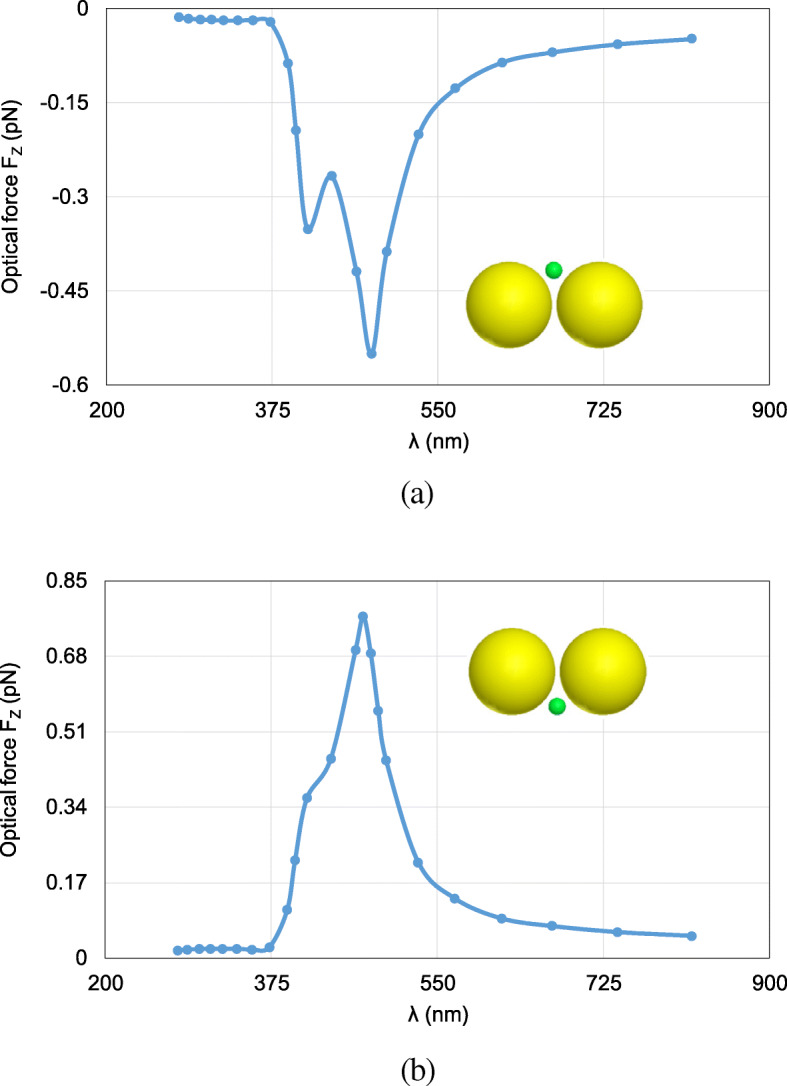
Calculated results of optical forces exerted on the nanosphere in the system of gold nanosphere dimer, varying with the wavelength *λ* of incident light. **a** The object nanosphere is located at (0, 0, 20 nm). **b** The object nanosphere is located at (0, 0, − 20 nm)

**Fig. 7:**
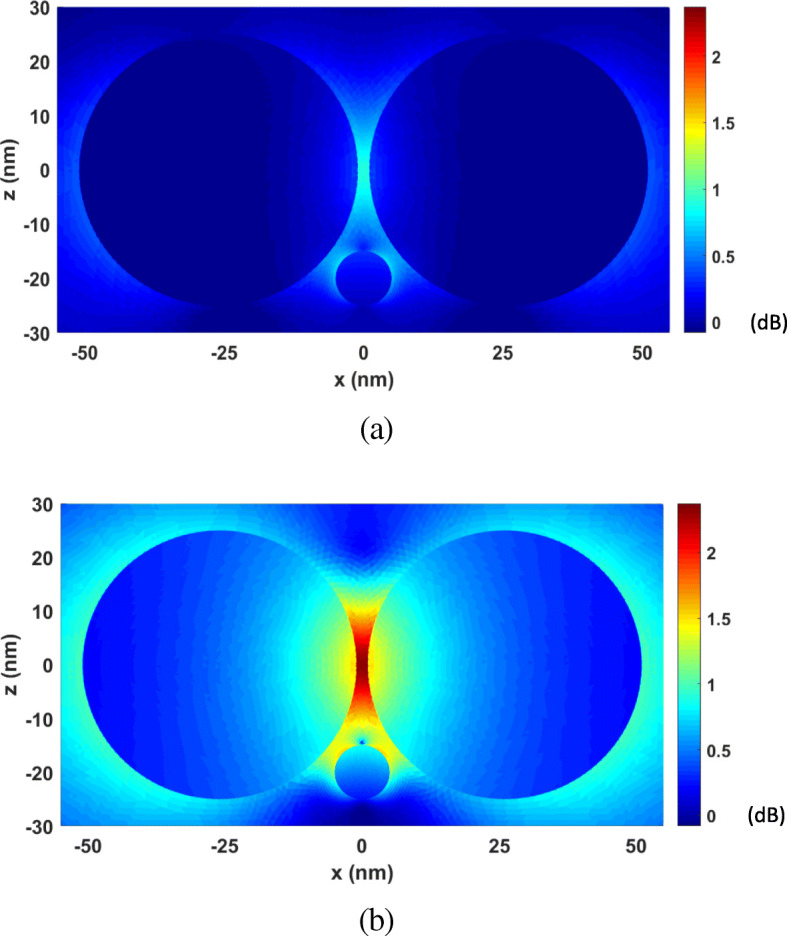
The electric field enhancement distributions on the *xoz* plane for the system of gold nanosphere dimer. **a** *λ* = 300 nm (non-resonance wavelength). **b** *λ* = 472 nm (resonance wavelength)

Next, the FETI-DP is tested for the optical force calculation with the wavelength *λ* varying from 277 nm to 818 nm. In practice, the analyses of optical force under incident light of different wavelengths are often necessary for searching the plasmonic resonance wavelength, where drastic field enhancement occurs and the strongest optical force can be obtained. Two cases are considered with the nanosphere located at (0, 0, 20 nm) and (0, 0, − 20 nm). Figure 6 a and b plots the calculated optical forces exerted on the nanosphere for different *λ*. It can be seen that the maximum optical force occurs at *λ* = 472 nm, which is the plasmonic resonance wavelength. The optical force at this resonance wavelength enhanced by nearly 40 times as against that at non-resonance wavelength. Moreover, the optical force always points to the dimer gap, as shown in Fig. 6, where the electric field intensity is strongest. It is also the direction of gradient force to trap the object. Figure 7 a and b shows the calculated electric field enhancement distributions at the non-resonance wavelength of *λ* = 300 nm and the resonance wavelength of *λ* = 472 nm, respectively. It can be seen that the electric field intensity has been increased by almost 250 times due to the plasmonic resonance effect.

**Fig. 8:**
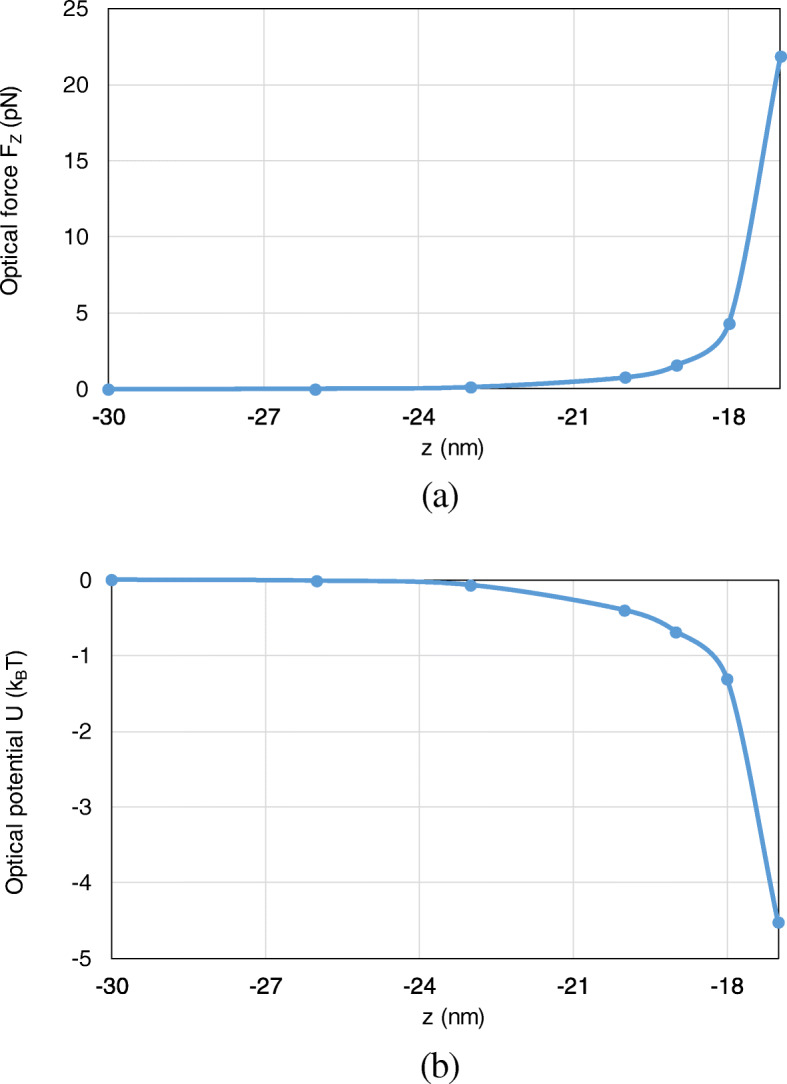
The optical forces and optical potentials exerted on the nanosphere in the system of gold nanosphere dimer, when the nanosphere moves from (0, 0, − 30 nm) to (0, 0, − 17 nm). **a** The optical forces. **b** The optical potentials

Besides, the optical force and optical potential are calculated with the nanosphere moving from (0, 0, − 30 nm) to (0, 0, − 17 nm) along the *z*-axis. Since the most typical and interesting behavior of trapping forces and potentials are those acting along *z*-direction, we here consider the axial trapping potential by integration along the *z*-axis. Because the motion of the nanoparticle is restricted to one dimension, the definition of an optical potential is unambiguous from (16), even though the total optical force from (15) is non-conservative. As shown in Fig. 8 a, b, with the nanosphere moving to the dimer gap, the optical force and optical potential depth obviously increase. At the position of (0, 0, − 17 nm), an optical potential depth of 4.6 *k*_*B*_T is produced, which is sufficient to overcome the Brownian motion in water to achieve stable optical trapping.

Finally, we test the effects of the dielectric substrate for this example. The optical forces are calculated with and without a substrate, respectively. For both two cases, the nanosphere is located at (0, 0, − 20 nm) and the incident wavelength is chosen as the resonance wavelength. For the case without substrate, the calculated result of the optical force is |**F**_0_| = 0.769 pN. For the case with a substrate, the gold nanosphere dimer is put on a dielectric substrate with a thickness of 60 nm and a relative permittivity of *ε*_*r*_ = 2.25. The calculated result of the optical force is |**F**_1_| = 0.761 pN. The relative error between these two results of optical forces is about 1.0 × 10^−2^, which is defined as |**F**_1_ − **F**_0_|/|**F**_0_|.

### Gold Truncated Cone Dimer

**Fig. 9:**
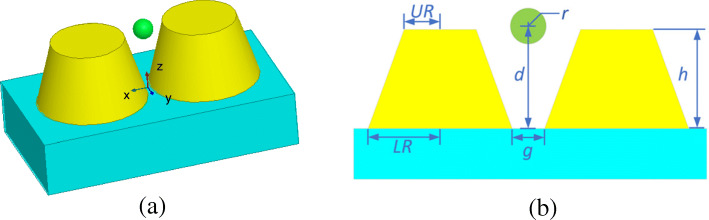
Configuration of an optical trapping system of a gold truncated cone dimer based on a dielectric substrate in water. **a** 3D view. **b** Front view and dimensions, where *UR* = 20 nm, *LR* = 30 nm, *h* = 35 nm, and *g* = 2 nm

The third example deals with the optical trapping of a gold nanosphere by using a gold truncated cone dimer. Figure 9 gives the configuration and dimensions of this system. The constitutive parameters of gold are taken from [32]. The dielectric substrate has a relative permittivity of *ε*_*r*_ = 2.25. The surrounding medium is water with a relative refractive index of *n* = 1.33. The incident light is plane wave with the power of 10 mW/μm^2^, the electric field polarization direction is +*x*, and the incident direction is −*z*. The whole computational domain is divided into 32 subdomains and discretized by tetrahedral meshes, which leads to 3.1 × 10^6^ unknowns, including 1.3 × 10^5^ dual unknowns and 1227 corner unknowns.

**Fig. 10:**
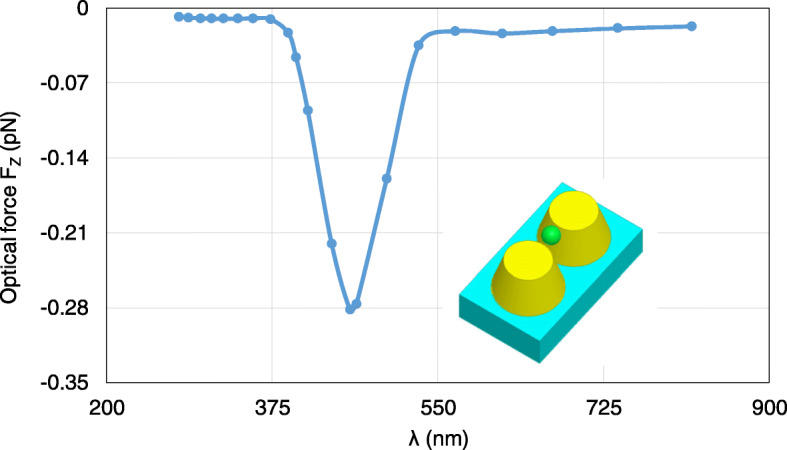
Calculated results of optical forces exerted on the nanosphere in the system of gold truncated cone dimer, varying with *λ*. The nanosphere is located at (0, 0, 35 nm)

**Fig. 11:**
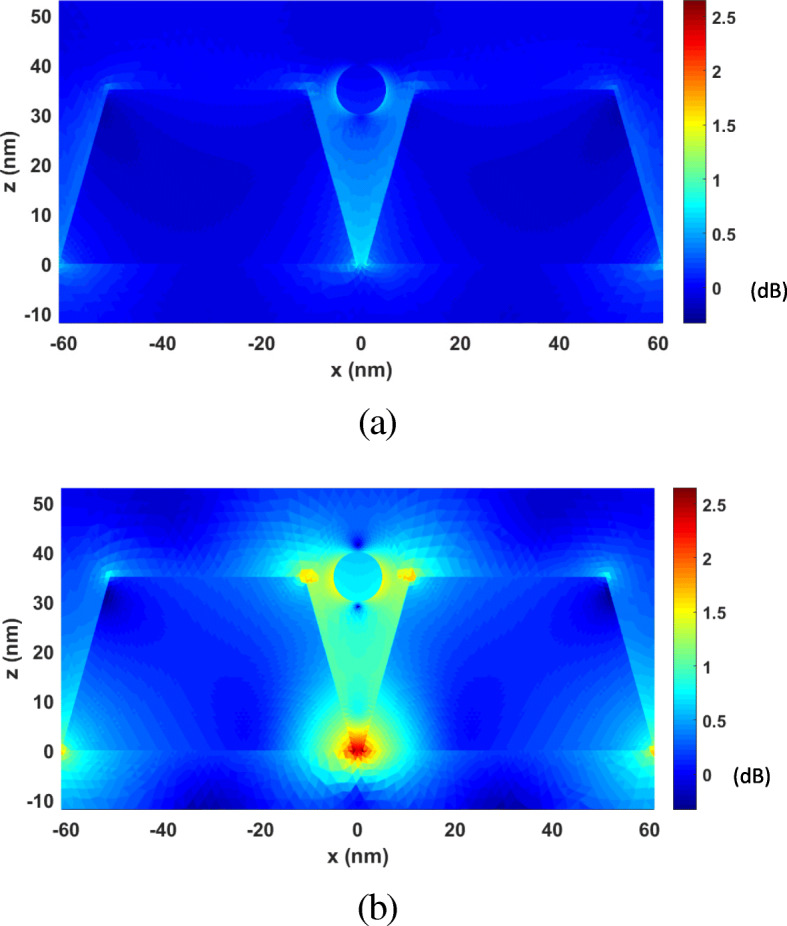
The electric field enhancement distributions on the *xoz* plane for the system of gold truncated cone dimer. **a** *λ*= 300 nm (non-resonance wavelength). **b** *λ*= 464 nm (resonance wavelength)

First, we analyze the optical forces by changing *λ* from 277 nm to 818 nm. Figure 10 plots the calculated optical forces exerted on the nanosphere for different *λ*. The nanosphere is located at (0, 0, 35 nm). It can be seen that the maximum optical force occurs at *λ* = 464 nm, which is the plasmonic resonance wavelength, and the optical force here is enhanced by nearly 30 times at non-resonance wavelength. Moreover, the total optical force always points to −*z*, as shown in Fig. 10, which is the direction of the gradient force. This confirms that the gradient force is greater than the scattering force, which is one of the conditions that the nanosphere can be stably trapped. Figure 11 a and b presents the calculated electric field distributions at the non-resonance wavelength of *λ*=300 nm and the resonance wavelength of *λ* = 464 nm, respectively. It can be seen that electric field intensity has been increased by almost 500 times due to the localized surface plasmon resonance.

**Fig. 12:**
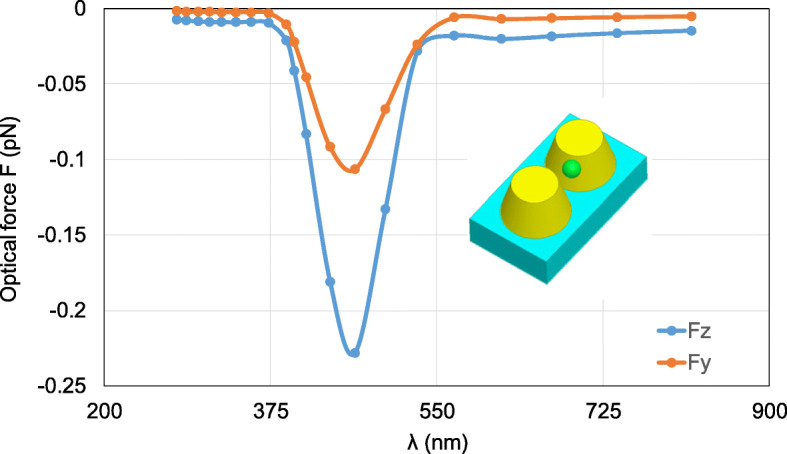
Calculated results of optical forces exerted on the nanosphere in the system of gold truncated cone dimer varying *λ*. The nanosphere is located at (0, 5 nm, 35 nm)

Then, the location of the nanosphere is changed to 0, 5, and 35 nm to observe the optical force. Figure 12 gives the calculated optical forces exerted on the nanosphere, where obvious *y*-component of optical force can be observed, while greater *z*-component of optical force exists. The total optical force still points to the position with the strongest electric field to trap the nanosphere.

**Fig. 13:**
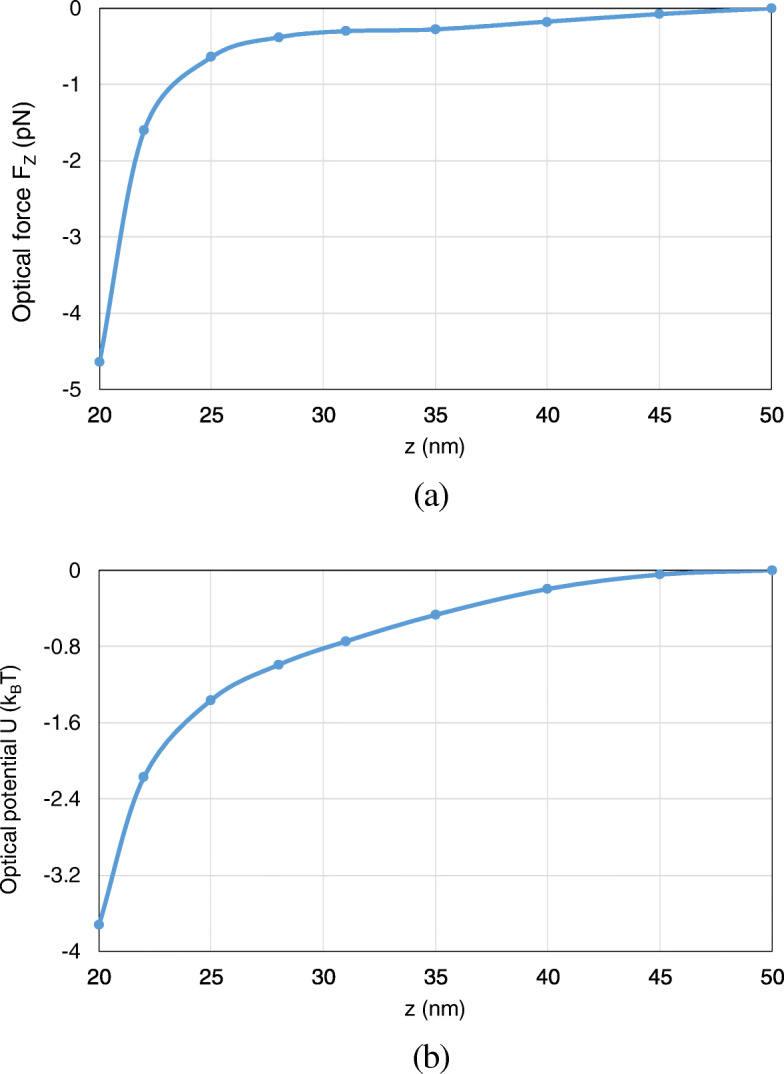
The optical forces and optical potentials exerted on the nanosphere in the system of gold truncated cone dimer, when the nanosphere moves from (0, 0, 50 nm) to (0, 0, 20 nm). **a** The optical forces. **b** The optical potentials

Furthermore, we analyze the optical potential with the nanosphere moving from (0, 0, 50 nm) to (0, 0, 20 nm) along the *z*-axis. Here, we consider the axial trapping potential along *z*-direction, which restricts the motion of the nanoparticle to one dimension and leads to an unambiguous definition of optical potential. Both the optical force and potential are calculated. As can be observed from Fig. 13 a, b, with the nanosphere moving to the dimer gap, the optical force and the optical potential depth obviously increase. At (0, 0, 20 nm), an optical potential depth of 3.8 *k*_*B*_T is obtained, which is sufficient to overcome the Brownian motion in water to achieve stable optical trapping.

Finally, we test the computational costs of the FETI-DP by changing the number of unknowns from 1.0 million to 3.2 million based on different mesh size. In practice, the tests under different mesh density are usually necessary to meet different accuracy requirements. Such a large-scale complex problem brings great challenges to conventional numerical methods. However, the FETI-DP can easily handle this problem. Thirty-two processors are employed for the FETI-DP simulation, while each processor deals with a subdomain. Table 3 reports the computational costs of the FETI-DP. It can be seen that the FETI-DP exhibits high simulation efficiency and low memory requirement.

**Table 3:** The computational costs of the FETI-DP for calculating the optical force in the system of gold truncated cone dimer. Thirty-two subdomains and 32 processors are used

Number of unknowns	Total memory (GB)	Total time (s)
1.0 × 10^6^	12.5	67.9
2.2 × 10^6^	29.0	180.1
3.2 × 10^6^	56.3	252.0

## Conclusion

An FETI-DP method combined with low-rank sparsification is proposed for the prediction and analysis of optical trapping of metal nanoparticles. The proposed method provides fully decoupled subdomain problems, which converts a large-scale complex problem into a series of small-scale simple problems. It is well-suited for parallel computation and can significantly improve the efficiency of numerical simulation. Examples demonstrate that the proposed method exhibits excellent performance of large-scale computation and is well-suited for the fast and accurate simulation of optical trapping at nanoscale.

## Data Availability

All data generated or analyzed during this study are included in this article.

## Abbreviations

ABC: Absorbing boundary condition
DOF: Degrees of freedom
FDTD: Finite-difference time-domain
FEM: Finite element method
FETI-DP: Dual-primal finite element tearing and interconnecting
MST: Maxwell stress tensor
MVP: Matrix-vector product
